# Historical and Generational Forces and Gender Differences in the Iridescent Life Course of Transgender Older Adults

**DOI:** 10.1093/geroni/igaa057.2613

**Published:** 2020-12-16

**Authors:** Charles Emlet, Karen Fredriksen Goldsen, Vanessa Fabbre, Hailey Jung, Hyun Kim

**Affiliations:** 1 University of Washington, Tacoma, Tacoma, Washington, United States; 2 University of Washington, Seattle, Washington, United States; 3 Washington University in St. Louis, St. Louis, Missouri, United States

## Abstract

Shifting historical, political, and social times have impacted transgender older adults in experiences and development. Examining how gender identities interact with life events within a historical context elucidates differences by generations and gender. Using a transgender subsample (n=205) from the National Health, Aging, and Sexuality/Gender Study (N=2,450), (logistic) weighted regressions with sociodemographic adjustment were performed to examine generational differences and gender interaction in key life experiences in identity, work, kin, bias, community, and health/well-being. Silenced (born 1935-1949, 18%), than Pride (1950-1964, 79%), Generation disclosed identity later, is less visible, more likely retired and veteran, more likely for lifetime opposite-sex marriage and divorce. Significant gender interactions exist in community engagement, kin relationships, and physical health. Despite small sample size, Invisible Generation (1914-1934, 3%) has unique differences. Findings show complexity of generational differences among transgender older adults, understanding of what helps better serve them as they age with their accumulated life experiences.

